# STRESS AND COPING AMONG NURSING ASSISTANTS AND PERSONAL CARE AIDES IN LONG-TERM CARE

**DOI:** 10.1093/geroni/igad104.1101

**Published:** 2023-12-21

**Authors:** Lea Efird-Green, Philip Sloane, Christine Lathren, Krista Perreira, Karen Bluth, Sheryl Zimmerman

**Affiliations:** University of North Carolina at Chapel Hill, Chapel Hill, North Carolina, United States; University of North Carolina, Chapel Hill, North Carolina, United States; University of North Carolina at Chapel Hill, Chapel Hill, North Carolina, United States; University of North Carolina at Chapel Hill, Chapel Hill, North Carolina, United States; University of North Carolina at Chapel Hill, Chapel Hill, North Carolina, United States; University of North Carolina at Chapel Hill, Chapel Hill, North Carolina, United States

## Abstract

Care in nursing homes and assisted living communities is largely provided by direct caregivers (nursing assistants [NAs] and personal care aides [PCAs]), who are >50% minoritized, 20% immigrant, 90% female, 87% without a college degree, and often in poverty. The well-being of direct caregivers is critical to quality of care, but research aimed at improving well-being is hampered because existing measures of stress and coping translate poorly to their backgrounds and work-related experience. To address this gap, we designed and conducted research aimed at describing the range of coping approaches used to address work-related stress, gathering data on a diverse sample of 391 direct caregivers from 9 nursing homes and 4 assisted living communities in 3 states, of which 75% were racially or ethnically minoritized and 21% were immigrant – mirroring the national workforce. Results indicated that 78% of respondents often felt stressed at work, with 30% screening positive for depression and 28% for anxiety, with some caregivers experiencing both (21%). However, working with residents and their families, while stressful (76%), was considered less stressful than workload (90%) and lack of support (83%); in fact, 89% of caregivers said that caring for residents makes them feel appreciated. Coping strategies were diverse, but the most common includedresilient coping (93%), mindfulness (89%), recognizing common humanity (84%), self-kindness (83%), and planning (69%). This presentation will address differences in coping based on demographic characteristics, as well as the relationship between types of coping strategies and reasons for staying in a direct caregiver position.

